# Editorial: Inorganic materials for energy and environmental applications

**DOI:** 10.3389/fchem.2022.977501

**Published:** 2022-07-22

**Authors:** Shuaifei Zhao, Qingyi Zeng, Chong-Chen Wang

**Affiliations:** ^1^ Institute for Frontier Materials, Deakin University, Geelong, VIC, Australia; ^2^ School of Resources and Environment and Safety Engineering, University of South China, Hengyang, Hunan, China; ^3^ Beijing Key Laboratory of Functional Materials for Building Structure and Environment Remediation, Beijing University of Civil Engineering and Architecture, Beijing, China

**Keywords:** heterogeneous catalysis, adsorption, energy conversion, pollutant removal, catalyst

**FIGURE 1:**
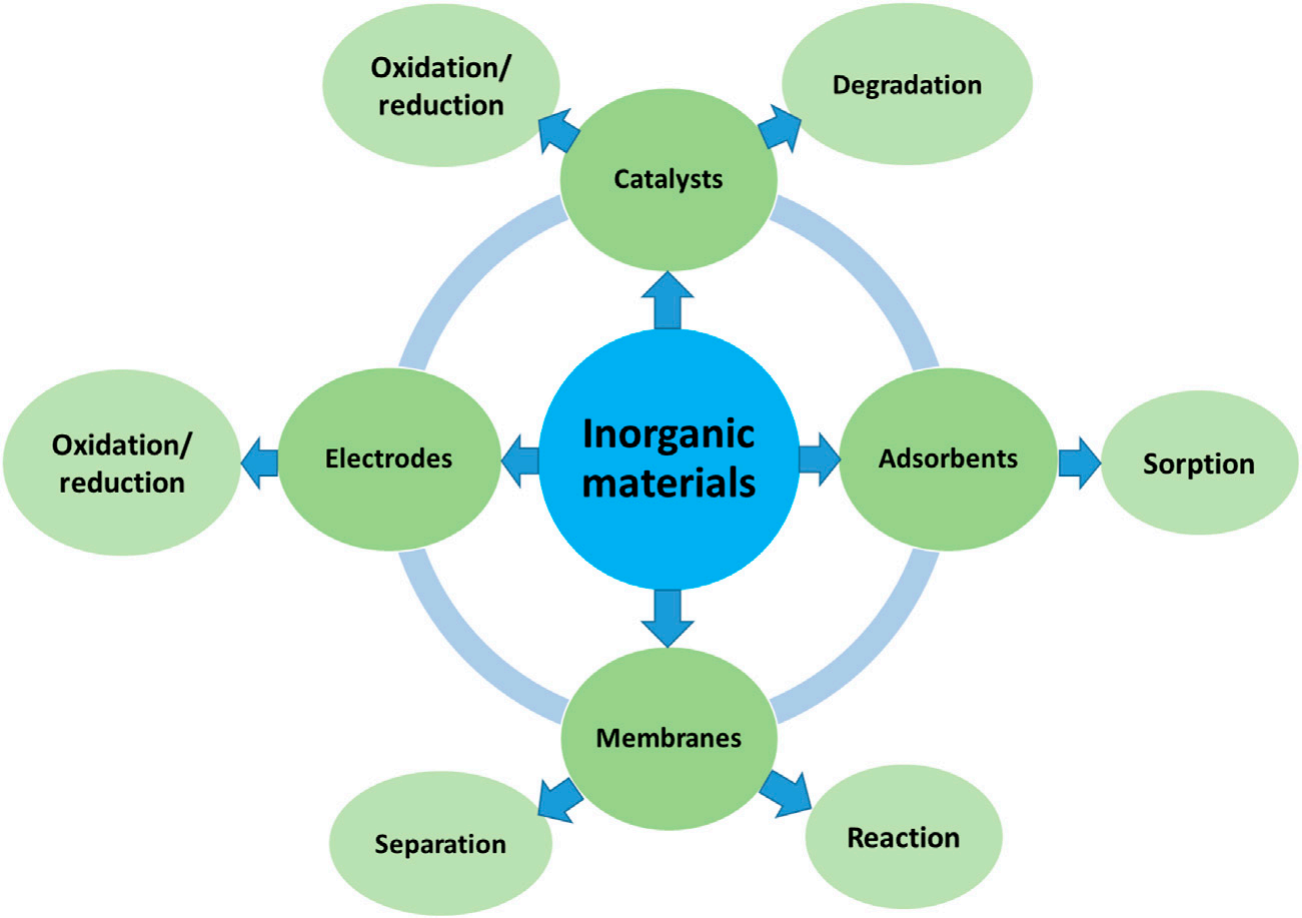
Typical applications of inorganic materials in the energy and environmental fields.

Inorganic materials have played significant roles in both energy conversion and environmental decontamination, relevant to chemical and environmental engineering. These inorganic materials are diverse, such as metals, metal oxides, nonmetallic oxides, sulphides, nitrides, phosphides and haloids. Inorganic materials often have high thermal stabilities, unique physicochemical properties and diverse nanostructures, making them highly desirable in various heterogeneous adsorption and catalytic applications (Goodman et al., 2020). As a result, they have been widely used as heterogeneous catalysts and/or adsorbents for CO_2_ conversion, fuel production, pollutant degradation or adsorption (He et al., 2020; Yang et al., 2021). Figure 1 summarizes the typical applications of inorganic materials in the energy and environmental fields, including as catalysts (Kuang et al., 2020), electrodes (Li et al., 2020), adsorbents (Zito and Shipley, 2015) and membranes (Song et al., 2016) for energy conversion via oxidation/reduction, or environmental decontamination *via* adsorption, rejection, oxidation or reduction (Zeng et al., 2020; Xu et al., 2021).

Numerous metals and metal oxides have been employed as electro- or photo-catalysts for energy conversion and pollutant degradation. Qi et al. developed a porous Ti_4_O_7_ electrocatalytic membrane by employing Ti powder as the reducing agent to thermally reduce TiO_2_ in vacuum. The prepared electrocatalytic membrane showed a high oxygen evolution potential (∼2.7 V/SHE). Wang et al. develop a new iron cathode electro-Fenton process coupled with a pH-regulation divided electrolysis cell for p-nitrophenol degradation. In the electrochemical Fenton system, an iron plate was used as the cathode to inhibit the release of iron ions and promote the reduction of Fe^3+^ to Fe^2+^. Therefore, excellent electrocatalytic degradation performance towards organic pollutants was achieved. Wang and Wang synthesized a NiO modified BiVO_4_ nanocomposite by a hydrothermal and calcination method. The as-prepared nanocomposite showed enhanced photoelectrochemical performance due to the unique NiO lamellar structure that provided a large number of active sites.

Wang et al. used a sol-gel self-combustion method to prepare carboxylate-rich carbon-modified Fe_3_O_4_ magnetic catalysts for heterogeneous Fenton degradation of organic pollutants. The prepared Fe_3_O_4_-based catalysts displayed improved heterogeneous Fenton degradation performance due to the enhanced pollutant adsorption. Zhu et al. synthesized a CdS/microcrystalline cellulose nanocomposite photocatalyst using an ultrasonic-assisted method. The prepared nanocomposite photocatalyst displayed enhanced pollutant degradation performance under visible light due to the heterojunction formation that efficiently separates the photogenerated electrons and holes of the photocatalyst. Wang et al. prepared a Co_3_O_4_/Ti cathode by electrodeposition for electrocatalytic reduction of nitrate, in which the NO_3_^−^ was reduced to N_2_ and NH_4_^+^ by the catalysis of Co_3_O_4_/Ti, and then NH_4_^+^ was selectively oxidized into N_2_ assisted by chloride ions and using IrO_2_-RuO_2_/Ti as the anode. Qiu et al. prepared Pt-modified TiO_2_ nanotubes as catalysts for photocatalytic degradation of Rhodamine B (RhB) under UV light. It was reported that the superoxide radical anions (O_2_^−^ **∙**), photogenerated hole (h^+^) and hydroxyl radical (OH**∙**) were the main active species contributing for RhB degradation.

In addition, metal and metal oxide based or modified materials have also been used for other catalytic applications. Bai et al. reported the Fischer–Tropsch synthesis performance of Co-based catalysts supported on graphitized ordered mesoporous carbon. The high catalytic performance resulted from the highly crystallized graphitic structure of the mesoporous carbon and the uniform dispersion of CoO on the support. Dai et al. used ion-exchange, *in situ* modification and complexation-excessive impregnation modification methods to modify SAPO-11 molecular sieves with Ni. The Ni-modified SAPO-11 molecular sieves were supported by NiWS catalysts for hydroisomerization of n-Hexadecane. The complexation-excessive impregnation modification method led to the best hydroisomerization performance. Huang et al. studied the effect of Ga_2_O_3_ on the hydrodesulfurization performance of 4,6-dimethyldibenzothiophene catalyzed by the stepwise impregnation method. Ga_2_O_3_ promoted Ni and Mo species to disperse uniformly and doping of more Ni atoms into the MoS_2_ crystals, increasing the average stacking number and the length of MoS_2_. As a result, enhanced hydrodesulfurization performance was achieved due to the formation of more NiMoS active phases in the system.

Adsorption is a simple but effective way for environmental decontamination (Zhang et al., 2018; Samadi et al., 2021). Various inorganic materials have been used for contaminant removal by adsorption. Zhang et al. prepared a series of nanostructured Fe-Cu binary oxides for arsenic removal. The crystallinity and structure of the Fe-Cu binary oxides had a significant impact on the arsenic adsorption performance. The oxides with lower crystallinity showed higher surface hydroxyl density and better adsorption performance. Li et al. reviewed the preparation, classification and applications of templated materials, particularly adsorbents in wastewater treatment. The templating method can endow materials with high specific area and unique porous structures, thereby enhancing the material sorption performance towards aqueous pollutants. Wei et al. reviewed the composite adsorbents for fluoride removal, including the adsorbent types (i.e., metal oxides/hydroxides, biopolymers, carbon-based, and others), preparation and sorption performance. The adsorption mechanisms for fluoride removal involving electrostatic attraction, ion exchange, complexation, and hydrogen bonding were also discussed.

Recently, with the promotion of the circular economy, waste based materials have attracted growing interest for various applications, such as fertilizers (Ye et al., 2019), carbon capture (Ji et al., 2018), membrane separation (Ni et al., 2022). Yu et al. prepared new biochar from excess sludge, followed by acetic acid modification. The modified sludge-derived biochar displayed improved porosity and enriched–COOH functional groups, thereby enhancing its adsorption performance to uranium. However, the catalytic performance of the sorbent was not discussed. Zeng et al. fabricated porous glass-ceramics based on coal fly ash without using pore forming agents by direct overfiring, in which borax was used to destroy the structure of quartz and amorphous vitreous body in coal fly ash and thus reduce the sintering temperature by the B-O bond. Chen et al. fabricated a non-sintered ceramsite from pyrite tailings for phosphorus removal. Both Plackett-Burman Design (PBD) and Box-Behnken Design (BBD) based response surface methodology were used to optimize the fabrication parameters.

Cellulase plays a key role in the production of fuel ethanol by enzymatic hydrolysis of lignocellulose, and immobilization of cellulase on the nanocarriers is an effective way to improve the hydrolysis efficiency. Wang et al. reviewed the significant roles of surfactants in oriented immobilization of cellulase on nanocarriers as well as a surfactant reversed micelle system.

In summary, this Research Topic discussed various inorganic materials as catalysts or adsorbents with unique nanostructures and functionalities for energy conversion and environmental decontamination. In the future, inorganic materials will continue to play a vital role in addressing global energy and environmental challenges, such as climate change, energy shortages and environmental pollution. Engineering new high performance heterogeneous catalysts and understanding the limiting factors and their mechanisms in the catalytic reaction are two key research directions that should be paid more attention to.
